# Genome-wide identification of GhRLCK-VII subfamily genes in *Gossypium hirsutum* and investigation of their functions in resistance to Verticillium wilt

**DOI:** 10.1186/s12870-023-04435-0

**Published:** 2023-09-11

**Authors:** Xiuyan Liu, Zhongping Lei, Yuzhen Yang, Zhenkai Wang, Shengying Ha, Zhangying Lei, Daohua He

**Affiliations:** 1 College of Agronomy, Northwest A&F University, No.3 Taicheng Road, Yangling, 712100 Shaanxi China; 2 College of Life Sciences, Northwest A&F University, Yangling, 712100 Shaanxi China; 3 Eighth Company of Rocket Farm, Xinxing, 839000 Xinjiang China

**Keywords:** *Gossypium hirsutum*, Receptor-like cytoplasmic kinases (RLCK)-VII, Gene expression analysis, Verticillium wilt, Virus-induced gene silencing (VIGS)

## Abstract

**Background:**

The receptor-like cytoplasmic kinases subfamily VII (RLCK-VII) is critical in regulating plant growth, development, and pattern-triggered immunity. However, a comprehensive exploration of these genes in the allotetraploid *Gossypium hirsutum* is still lacking. This study aimed to identify RLCK-VII genes in *G. hirsutum* and investigate their evolutionary history, structural features, expression patterns, and role in plant defense.

**Results:**

Seventy-two RLCK-VII genes in the *G. hirsutum* genome were unveiled and classified into nine groups following their phylogenetic analysis with *Arabidopsis thaliana*. Group VII-1 was the largest, accounting for 28%, while Groups VII-2 and VII-3 had only one member each. The analysis using MCScanX revealed that these 72 genes formed 166 collinear gene pairs and were resided on 26 chromosomes of *G. hirsutum*, suggesting that they were derived from whole genome segmental duplication events. Their calculated *Ka*/*Ks* values were below one, implying the occurrence of purification selection during the evolution and inhibition of gene function differentiation/loss. All members of the RLCK-VII subfamily possessed two conserved domains, PKinase-Tyr and PKinase, and several conserved PBS1 kinase subdomains, individually included in one of the ten motifs identified using MEME. The RNA-Seq results showed that RLCK-VII genes exhibited different spatiotemporal expression, indicating their involvement in cotton growth, development, and defense responses to *Verticillium dahliae*. The transcription patterns of RLCK-VII genes found by RNA-Seq were further validated using qRT-PCR assays after inoculating “20B12” (cotton cultivar) with “V991” (*V. dahliae*). The virus-induced gene silencing (VIGS) assays uncovered that two RLCK-VII genes (*Gohir.A13G227248* and *Gohir.A10G219900*) were essential to *G. hirsutum* resistance to Verticillium wilt.

**Conclusions:**

These observations offer valuable insight into the attributes and roles of RLCK-VII genes in *G. hirsutum*, potentially enable the breeding of new cotton cultivars with enhanced resistance to Verticillium wilt.

**Supplementary Information:**

The online version contains supplementary material available at 10.1186/s12870-023-04435-0.

## Background

Verticillium wilt (VW) is a destructive soil-borne plant illness caused by *Verticillium dahliae* infection, mainly damaging their vascular bundle system. The infection can cause cotton leaves to turn yellow, wilt, and even die, decreasing lint output and fiber quality and causing significant economic losses worldwide [1]. Developing new cultivars resistant to *V. dahliae* is the most productive and earth-friendly mean to manage the disease and support sustainable cotton production. However, no stable and durable cotton cultivars resistant to VW have yet been successfully developed. Therefore, identifying and characterizing genes associated with VW resistance using bioinformatic analysis and expression pattern analysis followed by function validation and genetic transformation will facilitate the creation of new cotton germplasms and cultivars with disease resistance.

Receptor-like kinase (RLK) is an important transmembrane protein in plants that made up of an extracellular receptor domain for signal perception, a single transmembrane region, an intracellular near-membrane domain, and cytoplasmic kinase domains, i.e., Pkinase (pfam00069) and Pkinase-Tyr (pfam07714), for signal transduction [2, 3]. The receptor-like cytoplasmic kinase (RLCK) is a special protein kinase family in the RLK superfamily and loses an extracellular signal sequence (ectodomain) and a transmembrane domain. Most RLCK proteins contain Ser/Thr intracellular kinase domains, and a minority of RLCK proteins also include other domains, such as U-box, leucine-rich repeat (LRR), pentatricopeptide repeat (PPR), WD40, and epidermal growth factor (EGF) [4]. *Arabidopsis* and rice have 147 AtRLCKs and 379 OsRLCKs members identified, respectively [4]. RLCKs in *Arabidopsis* are classified into 19 subfamilies based on phylogenetic relationships, referring to RLCK-II, RLCK-IV–RLCK-XVI, etc. [5]. Earlier explorations have uncovered that RLCK family genes are essential to plant growth, development, signal transduction, and response to biological and abiotic stresses [2, 4, 6, 7].

Remarkably, *Arabidopsis* RLCK subfamily VII genes have been comprehensively studied. A systematical identification indicated that *Arabidopsis* RLCK-VII subfamily has 46 members, including Botrytis-induced kinase 1 (BIK1), AvrPphB SUSCEPTIBLE 1 (PBS1), PBS1-Like1 (PBL1)–PBL43, and constitutive differential growth 1 (CDG1). Many RLCK-VII members play important roles in pattern-triggered immune (PTI) signaling [8]. Rao et al. [8] uncovered their shared and distinct functions in plant development and receptor kinases-mediated immunity. For example, BIK1, a member of the RLCK-VII subfamily of *A. thaliana*, effectively improves the immune response against *Botrytis cinerea* infection [9]. Latest studies have unveiled that BIK1 contributes to the pattern-triggered immunity (PTI) signaling pathway and co-activates PTI and effector-triggered immunity (ETI) signaling pathways to enhance plant immune responses. BIK1 phosphorylates RESPIRATORY BURST OXIDASE HOMOLOGUE (Rboh) D, a key molecular node that links PTI and ETI, leading to the formation of reactive oxygen species (ROS) that trigger plant immune responses, ultimately enhancing the plant immune response [2, 10, 11]. PBS1 and PBL1, which also belong to the RLCK-VII subfamily, can phosphorylate Rboh to produce ROS after the exogenous application of FLG22. These findings indicate that PBS1, PBL1, and BIK1 have redundant functions in the FLG22-mediated PTI immune response [12]. *Arabidopsis* receptor-interacting protein kinase (RIPK), i.e., AtPBL14, a member of the RLCK-VII subfamily, is critical to plant immune responses. AtRIPK activates AtRPMI receptor protein and phosphorylates AtRIN4 protein to improve plants’ resistance to *Pseudomonas syringae* pv. tomato DC3000 (Pst DC3000). The *ripk* mutants in *A. thaliana* reduce RIN4 phosphorylation and weaken the AtRIN4-mediated immune defense response [13]. While initially implicated in plant immune responses, RIPK mutants display a notable phenotype of reduced and shortened root hair. Studies have demonstrated that RIPK affects plant root hair development [12]. The *CaPIK1* gene, a member of the RLCK-VII subfamily with close phylogenetic relationship to *AtPBL5* or *AtPBL6*, is highly expressed in pepper, promoting ROS accumulation and enhancing immune ability against *Xanthomonas campestris* pv. vesicatoria. PIK1 gene silencing reduces resistance to pathogens [14].

Increasing reports on RLCK-VII subfamily genes in *A. thaliana*, rice, and other plants have shown that RLCK-VII genes may also participate in the response of *G. hirsutum* to *V. dahliae*. However, only a few studies have reported on RLK superfamily’s role in cotton, such as *GbRLK*, which is essential in regulating the drought and high salinity stresses pathway [15]. Dou et al. [16] identified 29 wall-associated kinases (*WAKs*) in *G. hirsutum* (*GhWAK*) genes, a group of the *RLKs*, uncovering the potential significance of *GhWAK* genes in cotton fiber cell development. Zhang et al. [17] identified 58, 66, and 99 *WAK/WAKL* genes in *G. arboreum*, *G. raimondii*, and *G. hirsutum*, respectively. Expression profiling analyses have shown that abiotic stresses induce *GhWAK/WAKL* expression. Furthermore, Feng et al. [18] unraveled a WAK-like kinase (*GhWAKL*) from upland cotton (*G. hirsutum*) and noted an elevated *GhWAKL* expression following *V. dahliae* infection and in response to salicylic acid (SA), implying that *GhWAKL* could serve as a promising molecular target for enhancing VW resistance in cotton. Other members, such as cysteine-rich receptor-like-kinases (*CRKs*), lectin receptor-like kinase (*GhLecRK-2*), and leucine-rich repeat-RLKs (*LRR-RLKs*), also contribute to the defense response [19–21]. Unfortunately, there is no reports specifically addressing RLCK-VII subfamily genes in *G. hirsutum*.

While studies of the expression patterns and regulation of RLCK-VII subfamily genes are crucial for genetic improvement and developing resistant cotton cultivars, a systematic investigation of RLCK-VII members in *G. hirsutum* has yet to be reported. Therefore, this study identified and characterized RLCK-VII subfamily members based on genome assembly and annotation combined with the transcriptome sequencing (RNA-Seq) dataset of *G. hirsutum*. Subsequently, we validated the expression profile using qRT-PCR and verified gene functions using virus-induced gene silencing (VIGS) assays. These findings offer novel insights into the molecular regulatory system of cotton defense responses against *V. dahliae* and may facilitate the breeding of resistant cotton cultivars.

## Results

### Identification and classification of RLCK-VII genes in *G. hirsutum* based on the phylogenetic tree

We identified genes encoding 4,513 proteins with the two conserved pfam00069 and pfam07714 domains in the *G. hirsutum* genome by using HMMER v3.3.1 software. Additionally, we found 949 proteins with an e-value < 1e^− 5^ by employing local BLASTP with the 46 amino acid sequences of *A. thaliana* RLCK-VII proteins as the queries. Because all these proteins were also included in the 4,513 proteins identified using HMMER software, they were further checked using SMART, NCBI CDD databases, DeepTMHMM, and local interProScan (Table S2). We classified proteins containing kinase domains but lacking transmembrane domains and ectodomains as members of the RLCK-VII subfamily, ultimately identifying 72 RLCK-VII genes in the *G. hirsutum* genome (Table 1).

**Table 1:** Physicochemical properties of RLCK-VII subfamily proteins in *G. hirsutum*

Gene_ID	Chr	Start (bp)	End (bp)	intron	AA	MW (Da)	pI	II	AI	GRAVY
Gohir.A01G021700	A01	2,117,396	2,122,579	4	463	51,085	6.72	35.74	71.64	-0.609
Gohir.A01G021800	A01	2,124,129	2,128,996	4	463	51,156	8.43	33.4	73.11	-0.629
Gohir.A01G078400	A01	11,524,454	11,527,243	4	389	43,207	5.05	38.59	76.5	-0.491
Gohir.A01G218000	A01	118,245,649	118,251,470	5	399	44,215	9.53	34.11	75.81	-0.495
Gohir.A02G085000	A02	33,038,821	33,042,584	5	423	46,400	9.29	26.04	75.15	-0.566
Gohir.A03G004500	A03	1,236,187	1,238,783	4	433	48,051	9.51	38.53	82.42	-0.328
Gohir.A03G022100	A03	4,314,869	4,322,158	6	398	44,844	6.28	38.58	87.71	-0.388
Gohir.A04G072800	A04	66,378,659	66,382,820	4	419	47,430	9.7	42.57	80.69	-0.501
Gohir.A05G034100	A05	3,525,039	3,527,403	4	451	51,230	9.17	41.72	79.33	-0.57
Gohir.A05G095300	A05	8,996,534	8,999,064	3	462	51,971	8.75	36.95	83.1	-0.413
Gohir.A05G095400	A05	8,999,473	9,003,237	6	404	44,631	9.53	36.41	75.15	-0.432
Gohir.A05G299000	A05	39,850,791	39,854,373	4	383	42,251	9.26	27.27	81.78	-0.426
Gohir.A05G364700	A05	101,335,119	101,339,515	5	492	54,431	9.49	43.27	69.8	-0.572
Gohir.A06G076700	A06	20,564,331	20,566,231	3	443	49,659	8.98	44.4	80.56	-0.477
Gohir.A06G147500	A06	116,496,281	116,498,458	4	387	43,288	5.97	35.42	86.18	-0.323
Gohir.A07G019000	A07	2,113,033	2,115,216	4	422	47,416	8.73	33.98	84.76	-0.375
Gohir.A08G000101	A08	171,259	174,163	4	424	47,042	8.04	37.76	74.53	-0.491
Gohir.A08G015600	A08	1,894,051	1,900,218	5	454	50,794	9.22	37.85	79.19	-0.573
Gohir.A08G028600	A08	3,483,004	3,486,007	5	355	39,666	8.27	33.81	88.7	-0.319
Gohir.A08G141200	A08	107,075,411	107,077,506	1	463	50,756	9.73	40.34	83.2	-0.351
Gohir.A08G161700	A08	113,231,423	113,234,231	3	389	43,768	9.51	39.14	77.15	-0.59
Gohir.A09G062200	A09	56,186,197	56,191,919	4	514	56,666	9.17	33.06	66.42	-0.696
Gohir.A09G107100	A09	67,742,321	67,749,970	5	411	45,303	9.3	26.2	77.59	-0.393
Gohir.A09G148400	A09	75,047,927	75,051,885	5	419	47,657	9.56	43.91	80	-0.498
Gohir.A10G014000	A10	1,160,042	1,172,744	4	388	43,074	6.77	28.15	91.44	-0.263
Gohir.A10G161100	A10	93,876,210	93,881,028	4	515	56,654	8.99	38.89	68.02	-0.706
Gohir.A10G204300	A10	111,109,457	111,113,134	3	429	49,065	9.71	42.53	82.03	-0.521
Gohir.A10G208100	A10	112,176,302	112,179,450	4	383	42,440	8.87	29.34	82.04	-0.409
Gohir.A10G219900	A10	114,535,031	114,538,442	5	386	42,797	9.12	35.7	76.55	-0.414
Gohir.A11G062700	A11	6,211,642	6,214,636	4	412	45,545	8.48	34.22	73.4	-0.496
Gohir.A11G278800	A11	114,254,706	114,256,725	4	377	42,652	8.29	41.5	92.52	-0.269
Gohir.A12G166000	A12	95,144,006	95,148,832	5	421	46,018	9.73	41.96	77.41	-0.388
Gohir.A13G072300	A13	18,726,335	18,729,980	5	420	45,843	9.54	40.84	79.43	-0.346
Gohir.A13G190500	A13	106,800,864	106,803,137	4	441	49,578	7.04	46.93	75.44	-0.569
Gohir.A13G207300	A13	109,008,577	109,014,372	5	440	49,150	6.58	36.73	72.73	-0.56
Gohir.A13G227248	A13	110,949,192	110,951,709	3	460	51,934	7.06	33.17	83.87	-0.39
Gohir.A13G227264	A13	110,952,241	110,955,399	6	402	44,552	9.4	35.06	76.17	-0.393
Gohir.D01G020200	D01	1,745,672	1,750,784	4	463	50,970	6.52	35.26	71.86	-0.594
Gohir.D01G089900	D01	15,927,003	15,931,452	5	381	42,290	8.58	38.5	82.91	-0.377
Gohir.D01G206900	D01	63,827,593	63,832,123	4	399	44,242	9.48	32.54	77.77	-0.461
Gohir.D01G211700	D01	64,296,311	64,299,766	5	371	41,388	9.54	41.97	80.7	-0.429
Gohir.D02G100700	D02	24,126,693	24,130,720	5	423	46,363	9.19	26.37	75.15	-0.565
Gohir.D02G164900	D02	62,097,630	62,100,471	6	381	43,074	8.85	46.23	86.22	-0.356
Gohir.D03G125400	D03	47,036,923	47,039,856	7	418	46,136	9.12	40.33	78.42	-0.379
Gohir.D03G146600	D03	51,021,330	51,028,940	6	399	44,896	6.61	37.33	88.72	-0.37
Gohir.D04G008100	D04	1,368,413	1,374,399	5	394	44,283	6.35	35.79	75.25	-0.583
Gohir.D04G050100	D04	8,457,130	8,461,583	5	492	54,424	9.35	42.4	69.59	-0.573
Gohir.D05G015700	D05	1,419,903	1,422,063	5	429	47,926	9.42	37.24	83.4	-0.47
Gohir.D05G035500	D05	2,978,868	2,981,060	4	451	51,242	9.07	40.22	78.47	-0.558
Gohir.D05G142100	D05	12,305,157	12,307,211	4	447	50,614	9.39	44.57	87.2	-0.478
Gohir.D05G298700	D05	32,843,967	32,847,589	4	383	42,298	9.2	27.68	81.51	-0.436
Gohir.D06G075700	D06	14,672,895	14,674,804	3	443	49,684	9.05	43.6	80.34	-0.481
Gohir.D07G041700	D07	4,587,533	4,591,224	5	427	47,588	9.29	33.57	78.99	-0.563
Gohir.D08G009100	D08	135,503	138,033	4	418	46,553	8.36	34.91	74.67	-0.493
Gohir.D08G162300	D08	52,977,997	52,980,359	1	463	50,727	9.64	39.33	85.94	-0.328
Gohir.D08G181700	D08	57,080,625	57,083,514	3	389	43,815	9.49	43	80.15	-0.567
Gohir.D09G039700	D09	14,877,151	14,880,805	5	426	47,420	8.25	25.22	78.31	-0.489
Gohir.D09G061100	D09	31,465,319	31,471,122	4	514	56,754	9.2	34.63	67.2	-0.697
Gohir.D09G103900	D09	39,406,325	39,410,857	5	411	45,275	9.3	26.6	77.37	-0.398
Gohir.D10G055900	D10	5,303,874	5,305,904	4	455	51,017	9.17	35.66	90.86	-0.395
Gohir.D10G212300	D10	60,851,172	60,854,916	4	429	48,987	9.71	42.53	82.73	-0.518
Gohir.D11G073300	D11	6,234,816	6,239,010	5	476	52,700	9.32	47.81	71.28	-0.543
Gohir.D11G079000	D11	6,694,616	6,697,779	5	438	49,407	9.37	40.98	78.84	-0.553
Gohir.D12G169400	D12	50,942,291	50,947,017	5	421	46,031	9.76	41.77	77.65	-0.374
Gohir.D12G214600	D12	57,064,063	57,066,630	3	386	43,117	9.73	40.05	81.32	-0.536
Gohir.D13G068300	D13	10,770,674	10,775,504	5	486	53,903	9.2	40.46	71.67	-0.586
Gohir.D13G072400	D13	12,416,197	12,419,884	5	420	45,827	9.54	40.94	78.5	-0.361
Gohir.D13G091900	D13	21,878,192	21,881,709	4	426	47,120	6	42.66	78.83	-0.51
Gohir.D13G211826	D13	62,269,833	62,275,738	5	426	47,696	8.4	37.3	73.97	-0.552
Gohir.D13G232700	D13	64,442,371	64,445,037	3	460	51,878	8.1	33.54	82.61	-0.41
Gohir.D13G232800	D13	64,445,481	64,448,651	6	402	44,442	9.43	32.97	75.92	-0.406
Gohir.D13G235200	D13	64,589,706	64,592,749	4	419	46,466	8.48	23.98	77.04	-0.466

Note: Physicochemical properties obtained by online software ProtParam (http://web.expasy.org/ProtParam/)

Chr: Chromosome; Start: Start position (bp); End: End position (bp); intron: number of introns; AA: Amino acid; MW: Molecular weight (Da); pI: Isoelectric point; II: instability index; AI: Aliphatic index; GRAVY: Grand average of hydropathicity

**Fig. 1:**
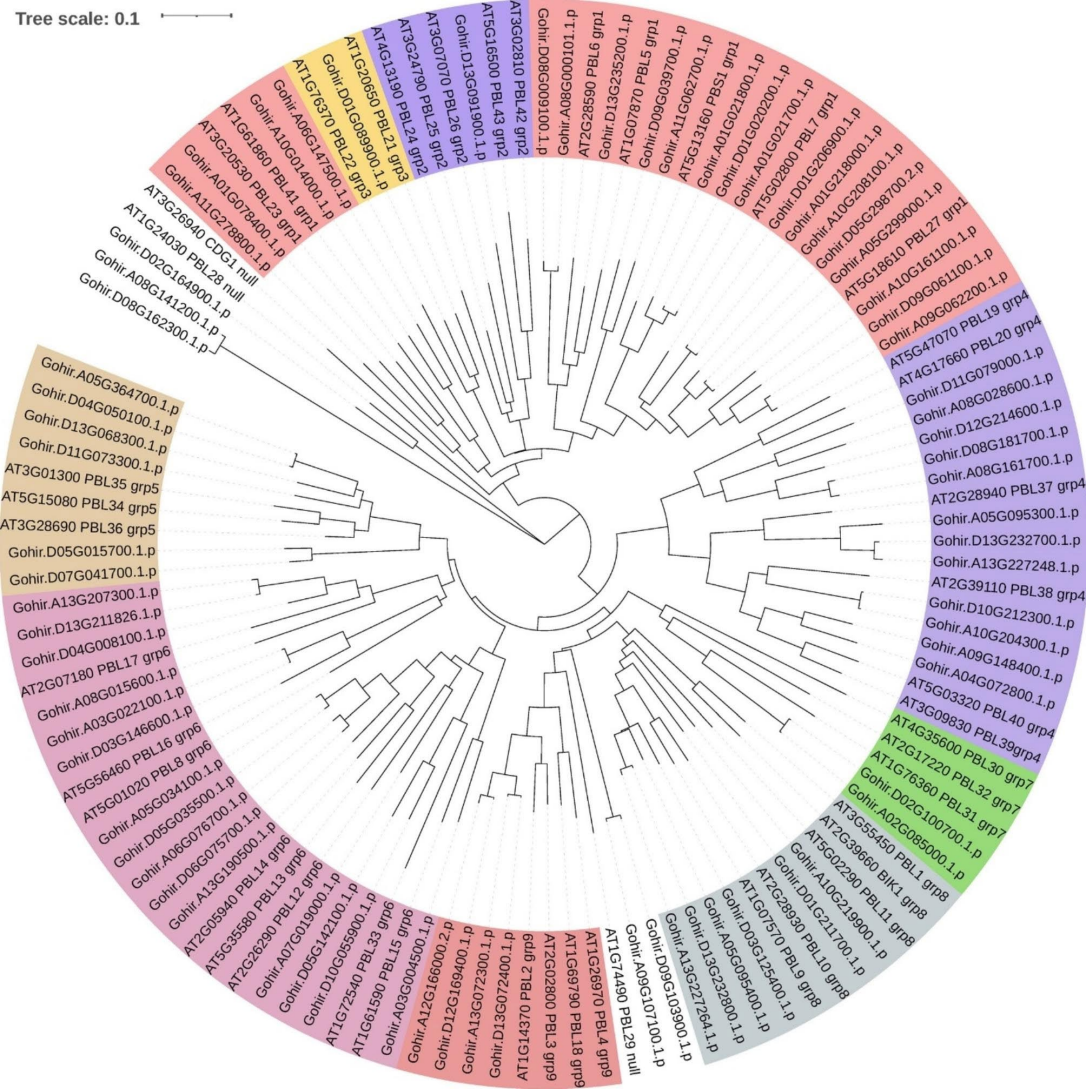
Phylogenetic tree depicting proteins encoded by 72 *GhRLCK-VII* genes in *G. hirsutum* genome and 46 *AtRLCK-VII* genes in *A. thaliana* genome

To classify the RLCK-VII genes in *G. hirsutum*, we generated a phylogenetic tree based on the amino acid sequences of 72 GhRLCK-VII members and 46 AtRLCK-VII members (Table S3, Fig. 1). According to the classification of AtRLCK-VII members, we classified the 72 GhRLCK-VII members into nine groups, named RLCK-VII-1 to RLCK-VII-9 [8]. We further divided the RLCK-VII-1 group into RLCK-VII-1-A and RLCK-VII-1-B. The quantity of members within each group varied, with RLCK-VII-1 being the largest (including 20 genes, accounting for 28%) and VII-2 and VII-3 being the smallest (each with only one member) groups. There were 12 genes in the RLCK-VII-4 group, 15 genes in the RLCK-VII-6 group, 2 genes in the RLCK-VII-7 group, 6 genes in each of the RLCK-VII-5 group and VII-8 group, and 4 genes in the RLCK-VII-9 group. In addition, 5 genes were not classified into any of the groups. The protein sequence distance (diversity) of GhRLCK-VII and AtRLCK-VII in each group ranged from 0.2945 to 0.6059. The classification of GhRLCK-VII and sequences similarity of GhRLCK-VII and AtRLCK-VII (in a model plant) provided clues for investigating the roles of GhRLCK-VII members.

### Prediction of chromosome location, physicochemical properties, signal peptide, and subcellular localization

**Fig. 2:**
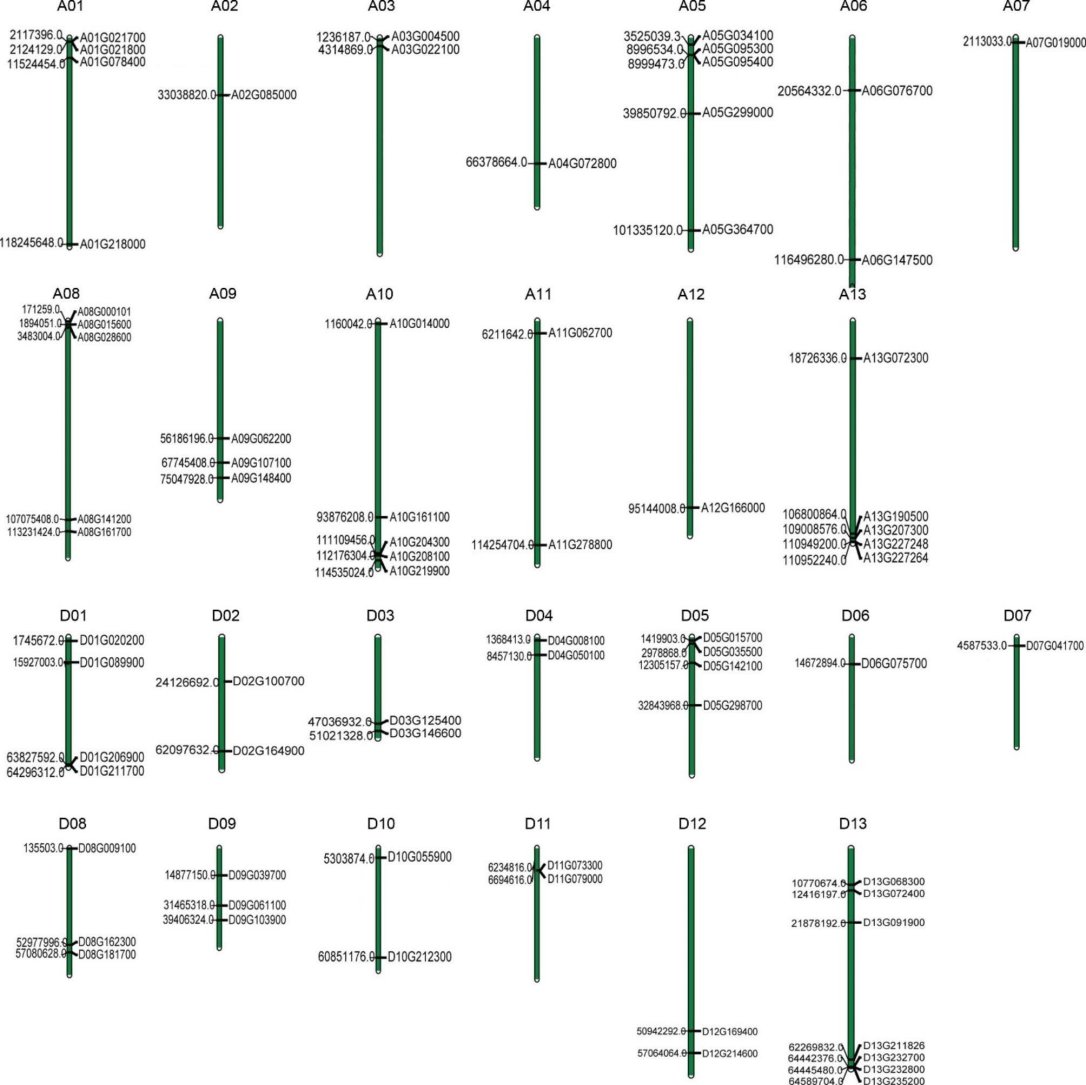
Localization of GhRLCK-VII subfamily genes on the 26 chromosomes in *G. hirsutum* genome

Based on genome information, 72 GhRLCK-VII genes were distinctly distributed on the 26 chromosomes of *G. hirsutum* (Fig. 2). The largest group, GhRLCK-VII-1, consisted of genes (20) distributed on 12 chromosomes, while six chromosomes (A02, A04, A07, A12, D06, and D07) each harbored only one gene. Chromosome D13 had the largest number (7) of GhRLCK-VII genes. GhRLCK-VII-5 and VII-8 each contained 6 genes distributed on 6 chromosomes. These data indicated that genes within one group were unevenly distributed on the 26 chromosomes.

The physicochemical parameters of GhRLCK-VII proteins were analyzed using ProtParam software (Table 1). Their amino acid residues were in the range from 355 (Gohir.A08G028600) to 515 (Gohir.A10G161100), with an average of 426. Forty-nine (68%) GhRLCK-VII proteins had 400−500 amino acid residues, 20 (27.8%) had fewer than 400 amino acid residues, and 3 (4.2%) with more than 500 amino acid residues. The number of amino acid residues was significantly different between GhRLCK-VII-5 and GhRLCK-VII-3 (*p* < 0.05). Their molecular weight ranged from 39,666.44 to 56,753.74 Da, with 51 (70.8%) proteins ranging from 41,388.09 to 49,683.9 Da. Most proteins in the GhRLCK-VII subfamily were basic proteins, with 86% of proteins having pI values > 7.0 and only 14% of proteins having pI values < 7.0. The protein instability index varied from 23.98 to 47.81, with a mean value of 37.1. The aliphatic index of GhRLCK-VII proteins was less than 100, in the range of 66.42–92.52, indicating that all 72 proteins had a wide range of temperature stability. The grand average of hydropathy (GRAVY) values were all negative, implying that all 72 GhRLCK-VII proteins were hydrophilic. Analysis using the SignalP server 6.0 revealed that GhRLCK-VII proteins had no signaling peptides. Subcellular localization analysis unveiled that all GhRLCK-VII proteins were intracellular proteins, with 80.5% of proteins located in the cytoplasm and 19.4% of proteins localized in the chloroplasts.

### Analyses of gene structures, protein domains, and motifs

**Fig. 3:**
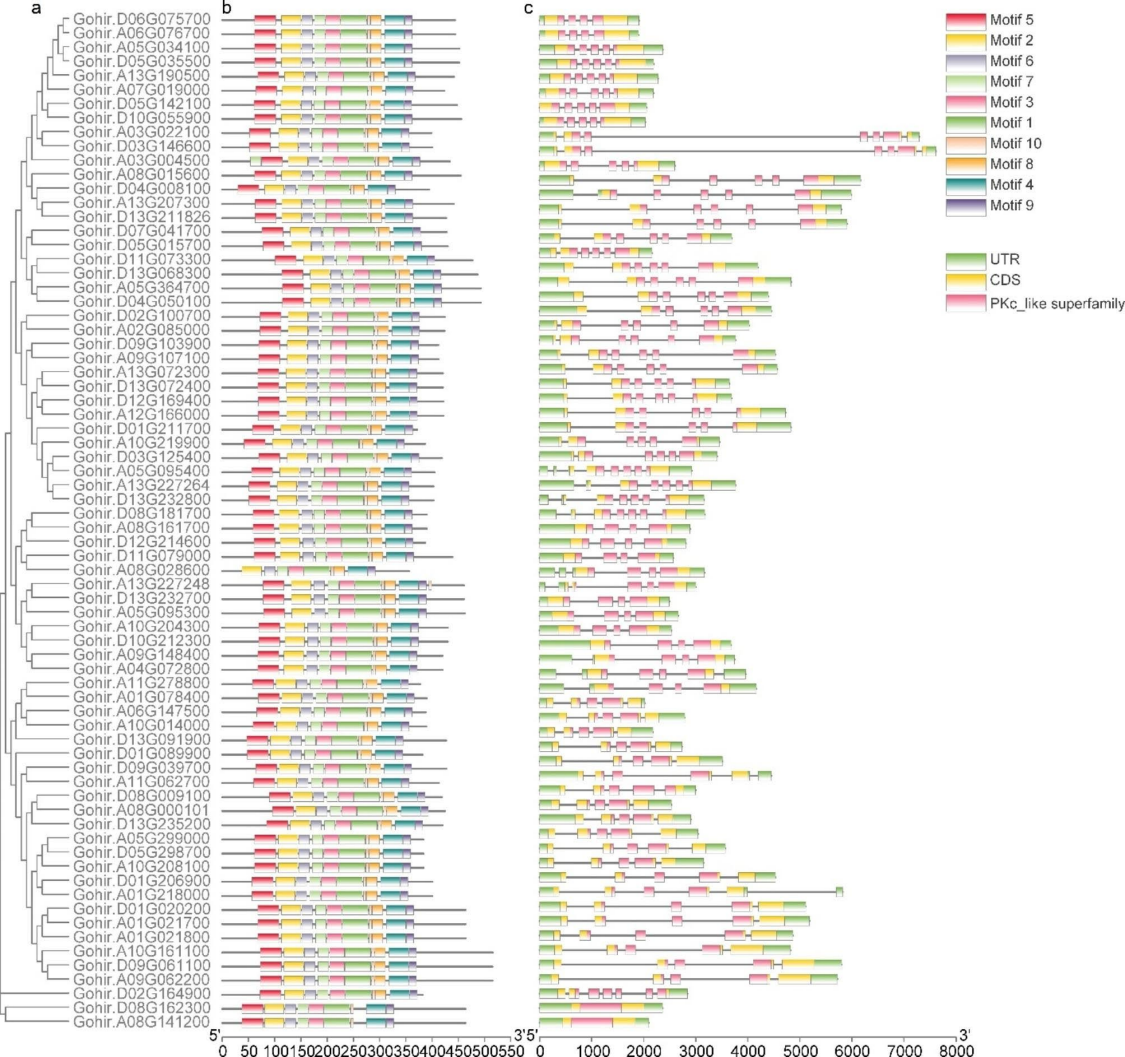
Phylogenetic relationship, motif architecture, gene structure (exon-intron organization), and conserved domains of GhRLCK-VII genes. **(A)** Phylogenetic tree. **(B)** Motif composition and distribution. The colored boxes indicate different conserved motifs identified using MEME. **(C)** Gene structure (exon-intron organization). The sequences of UTR, CDS, and PKc_like superfamily are represented in different colors

The conserved motifs of GhRLCK-VII proteins were unraveled using MEME. Motif-1 and motif-2, which are included in Pkinase_Tyr or Pkinase domains, were found to have conserved sequences. All 72 (100%) proteins contained motif-1 and motif-2, as shown in Table S4 and Table S5. Gohir.A03G004500 of the GhRLCK-VII-6 group contained an additional conserved motif-7, Gohir.A08G028600 of the GhRLCK-VII-4 group lacked motif-5, Gohir.A13G227248 of the GhRLCK-VII-4 group contained an extra motif-10, and Gohir.D08G162300 and Gohir.A08G141200 lacked a motif-8. Furthermore, 67 (93%) proteins of the GhRLCK-VII subfamily contained 10 conserved motifs. These findings showed similar motif arrangements within the same group, but slight diverse motif arrangements among different groups (Fig. 3).

To further understand the GhRLCK-VII subfamily genes, their “exon-intron” structures were investigated, and the diagrams showed similar gene structures (Fig. 3). The number of exons was 1 to 8, with 69 (96%) genes containing 4–7 exons. *Gohir.D03G125400* of the RLCK-VII-8 group contained the most (8) exons, whole *Gohir.A08G141200* and *Gohir.D08G162300*, which were not classified into any group, each had only two exons. The number, length, and location of exons/introns were comparable among genes in the same group, indicating that the 72 genes kept roughly conserved structures during evolution, with some divergences among different groups.

The GhRLCK-VII subfamily proteins contained two conserved domains, Pkinase-Tyr (pfam07714) and Pkinase (pfam00069), both of which were members of the PKc_like superfamily (cl21453). According to the results of NCBI CD-Search, all 72 proteins contained PKc_like superfamily domains (Fig. 3).

### Gene duplication and collinearity analysis of RLCK-VII subfamily

**Fig. 4:**
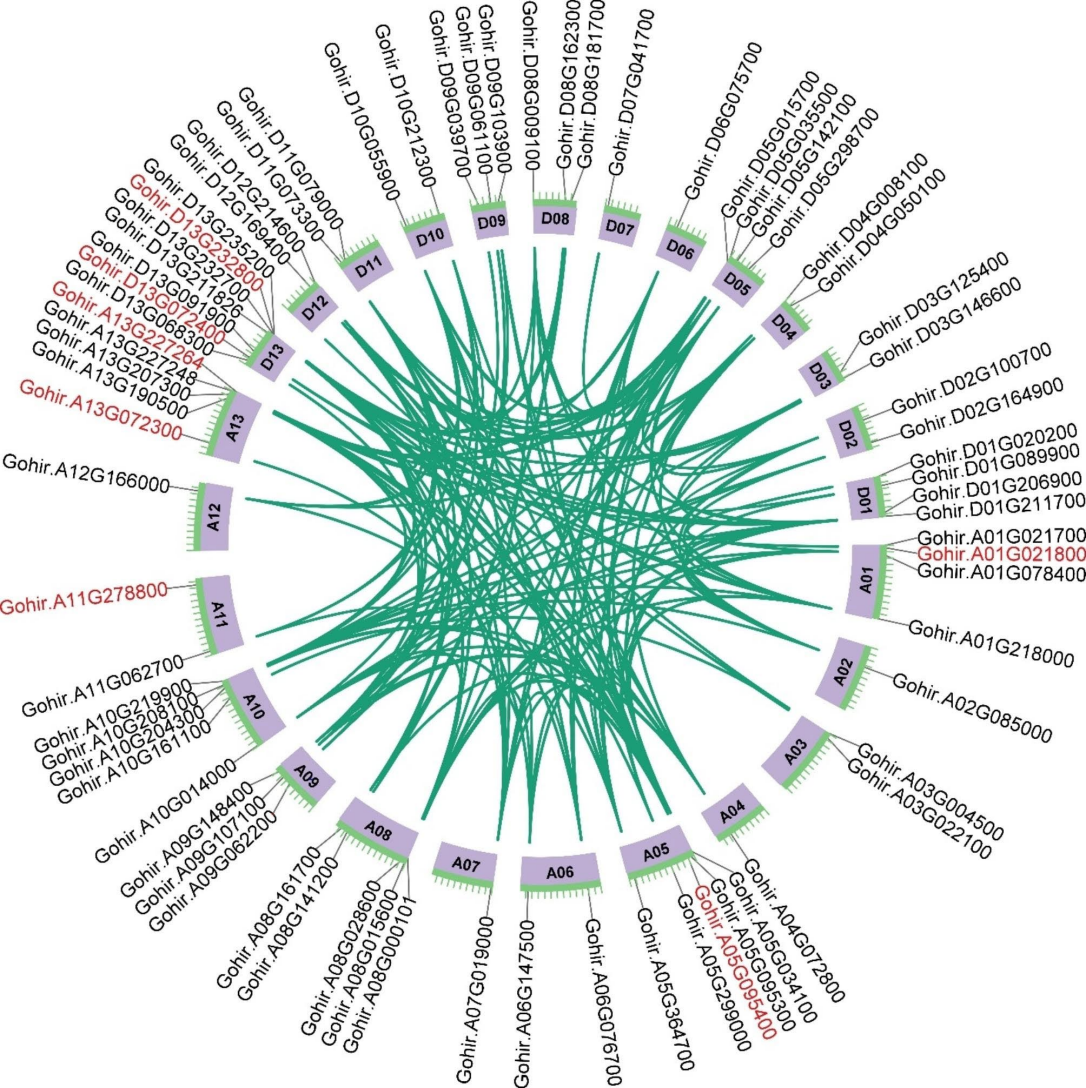
Collinear relationships of RLCK-VII genes, revealing segmental duplications of RLCK-VII genes and gene-pairs involved in segmental duplication. The red-colored genes represent the collinearity between RLCK-VII and other family genes, and the unmarked red genes represent the collinearity within the RLCK-VII family

To further understand the evolution of GhRLCK-VII subfamily, we investigated tandem duplication and segmental duplication events of GhRLCK-VII genes using MCScanX (Fig. 4). Our findings suggested that segmental duplication was the primary driver of subfamily’s expansion during evolution, with only six GhRLCK-VII genes involved in tandem duplication (Table S6). We found a total of 266 pairwise collinear relationships involving one gene (100 pairwise collinearity between 7 RLCK-VII genes and others) or two genes (166 pairwise collinearity among 65 genes) in the GhRLCK-VII subfamily. Of the 7 genes of the GhRLCK-VII subfamily involved in the 100 pairwise collinearity, none had GhRLCK-VII counterparts, suggesting that they might have been lost or undergone prolonged positive (adaptive) selection. We also observed 26, 24, and 28 pairwise collinear relationships among chromosomal regions containing genes in the RLCK-VII-1 group, in the RLCK-VII-4 group, and in the RLCK-VII-6 group, respectively. Additionally, we found 21 pairwise collinear relationships in subgenome A_t_ and 17 pairwise colinear relationships in subgenome D_t_, respectively, in addition to the collinear relationships between subgenome A_t_ and D_t_. Furthermore, we observed the collinearity of single genes corresponding to multiple genes in the GhRLCK-VII subfamily. These results indicated that the GhRLCK-VII subfamily underwent segmental duplication events during evolution, resulting in either one or several times of duplication events of certain chromosomal segments. Our collinearity analysis indicated that chromosomal segmental duplication contributed significantly to the expansion of RLCK-VII subfamily genes in *G. hirsutum* during evolution.

To investigate the evolutionary selection pressure on the GhRLCK-VII subfamily after chromosomal segmental duplication, the *Ka/Ks* ratios of collinear gene pairs was calculated using KaKs_Calculator. The results uncovered that 166 gene pairs involved in segmental duplication had *Ka/Ks* ratios less than 1, implying the occurrence of strict purification selection during evolution and severe inhibition of functional differentiation (Table S6). We inferred that the genes arising from segmental duplication were relatively conservative in evolution, preserving their previous protein structures and functions without losing biological activity, ultimately enhancing specific biological functions (such as stress tolerance) in plants.

### Identification of *cis*-acting regulatory elements in GhRLCK-VII gene promoters

**Fig. 5:**
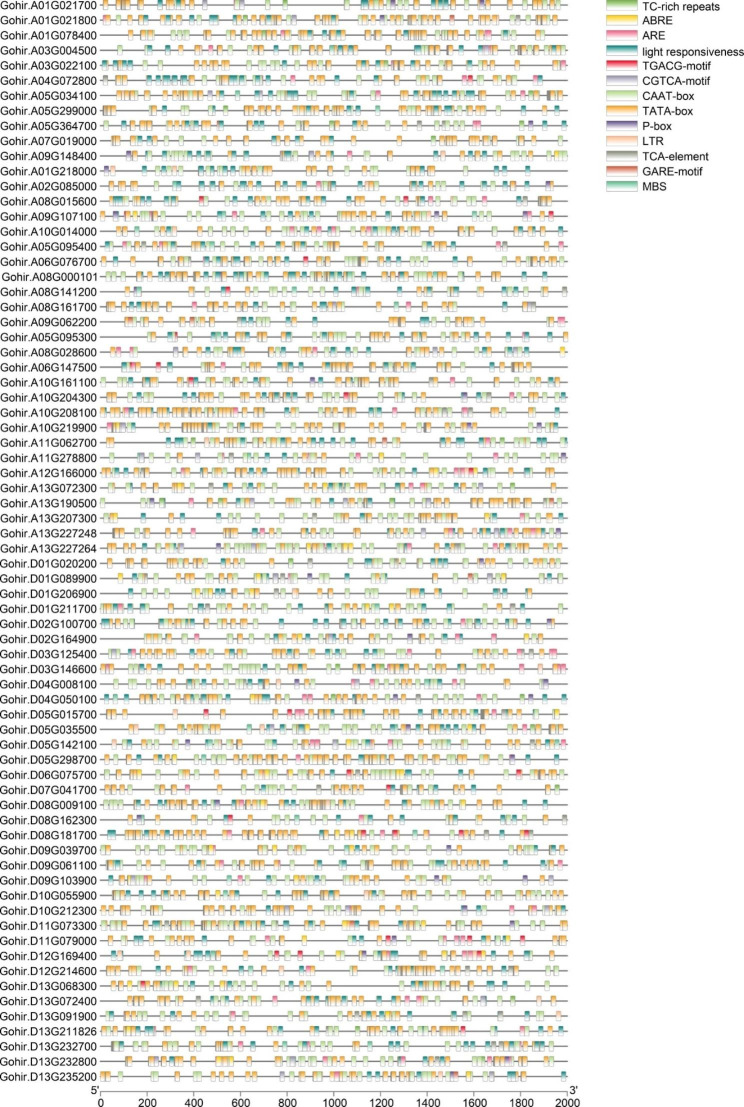
Predicted *cis*-elements in RLCK-VII promoters

The gene promoter region often possesses *cis*-acting regulatory elements linked to its function. We found 48 *cis*-acting elements using the program PlantCARE in the upstream region of GhRLCK-VII genes (Table S7). We focused on 11 important elements for visualization based on the characteristics of the RLCK-VII gene subfamily, excluding general transcription regulatory elements and unknown-functional elements (Fig. 5). We observed CAAT-box, TATA-box, and light response elements in the promoter region of 72 GhRLCK-VII genes. In addition, we found TC-rich repeats, low-temperature responsiveness region, and MYB binding site (MBS) in 28 genes, salicylic acid responsiveness element and TCA element in 41 genes, ABA-responsive element in 48 genes, anaerobic induction element ARE in 65 genes, methyl jasmonate (MeJA) reaction element in 46 genes, and gibberellin induction element in 37 genes. Most genes contained multiple *cis*-acting elements. These findings implied the involvement of GhRLCK-VII subfamily in plant growth, development, hormone response, and resistance to biological and abiotic stresses, provided insights into the gene expression regulation, and enhanced our understanding of GhRLCK-VII gene functions in plants.

### Expression profile analysis based on RNA-Seq datasets and qRT-PCR assays

**Fig. 6:**
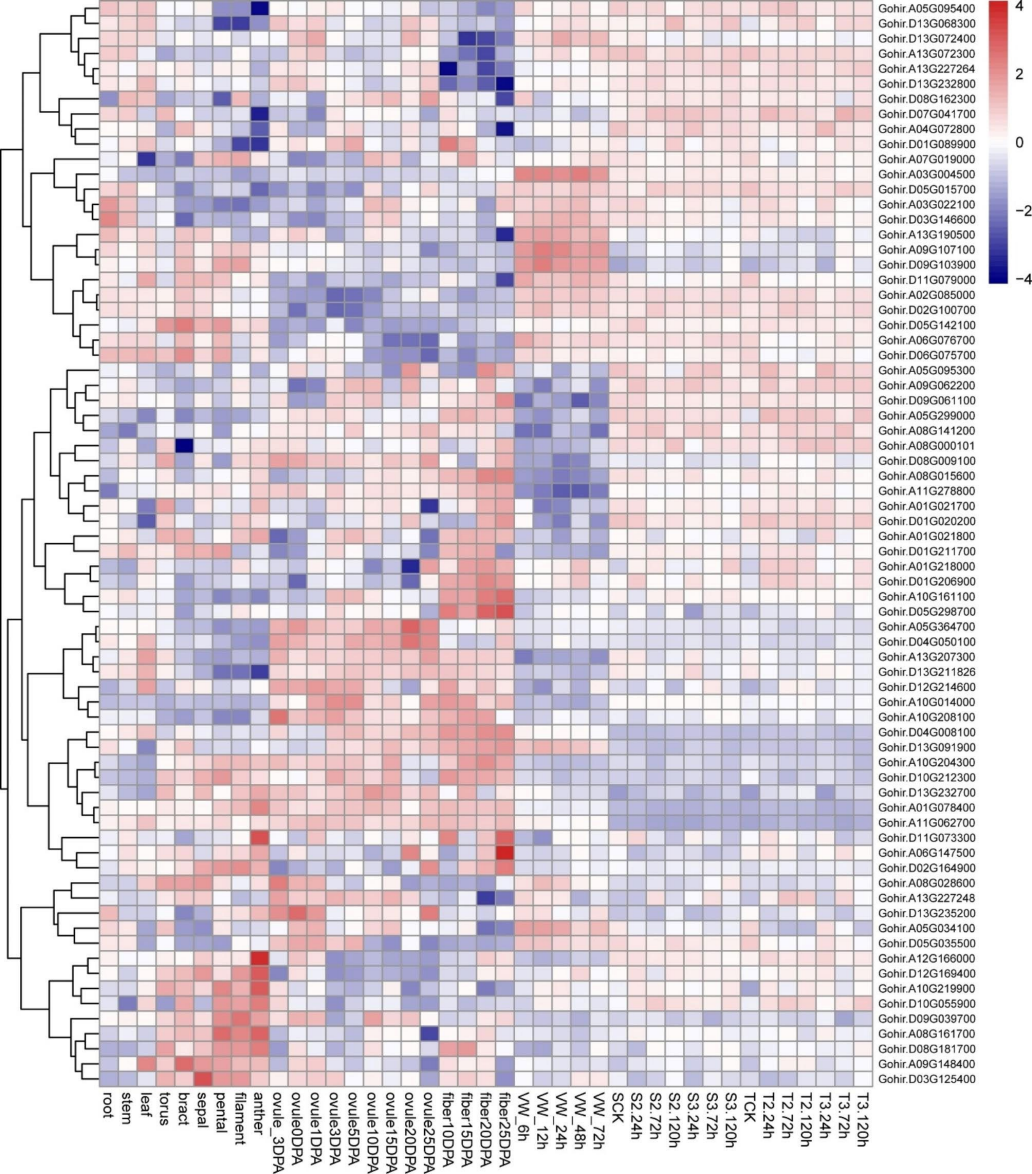
Expression profile of RLCK-VII genes based on three RNA-Seq datasets

To get insights into the expression patterns of GhRLCK-VII subfamily, we analyzed RNA-Seq datasets SRP166405 from various tissues of *G. hirsutum* and SRP192537 and SRP328396 after inoculation with *V. dahliae*. Our results demonstrated distinct expression patterns of GhRLCK-VII genes in different tissues and developmental stages of *G. hirsutum* (Fig. 6, Table S8), with *Gohir.A08G161700* being highly expressed in petals, filaments, and anthers, *Gohir.A09G148400* being expressed significantly higher than other genes in the bract, and *Gohir.D03G125400* being generally higher expressed in all tested tissues. Moreover, *Gohir.D10G212300* and *Gohir.D11G073300* genes were prominently expressed in petals and anthers, signaling their possible involvement in the growth and development of corresponding tissues, respectively. Moreover, some GhRLCK-VII genes, including *Gohir.A04G072800*, were down-regulated during ovule and fiber development, while others, including *Gohir.D03G146600* and *Gohir.A06G147500*, were up-regulated, suggesting their involvement in the growth and development of these tissues.

We also explored GhRLCK-VII gene expression in cotton inoculated with *V. dahliae* based on the RNA-Seq datasets SRP192537 and SRP328396. Our finding demonstrated that 72 genes were significantly up-regulated in *V. dahliae*-tolerant cultivar Zhongzhimian-2 by 58-fold at 24 h, 72 h, and 120 h of post-inoculation with *V. dahliae* (strain 2 and strain 3), but only changed 25-fold in *V. dahliae*-susceptible cultivar Xinluzao-36. Several genes, including *Gohir.D11G079000*, were differentially expressed only in Zhongzhimian-2 after *V. dahliae* infection, while other genes, including *Gohir.D13G232700*, were differentially expressed in both cultivars at some time-points. The up-regulation of *Gohir.D02G100700*, *Gohir.A13G227264*, *Gohir.A13G227248*, *Gohir.A10G219900* and *Gohir.A02G085000* and downregulation of *Gohir.A05G034100*, *Gohir.D05G035500*, and *Gohir.D11G079000* in Zhongzhimian-2 after *V. dahliae* inoculation suggested that the increased transcription of more GhRLCK-VII genes in Zhongzhimian-2 than in Xinluzao-36 contributed to the tolerance of Zhongzhimian-2 to *V. dahliae* (Fig. 6).

**Fig. 7:**
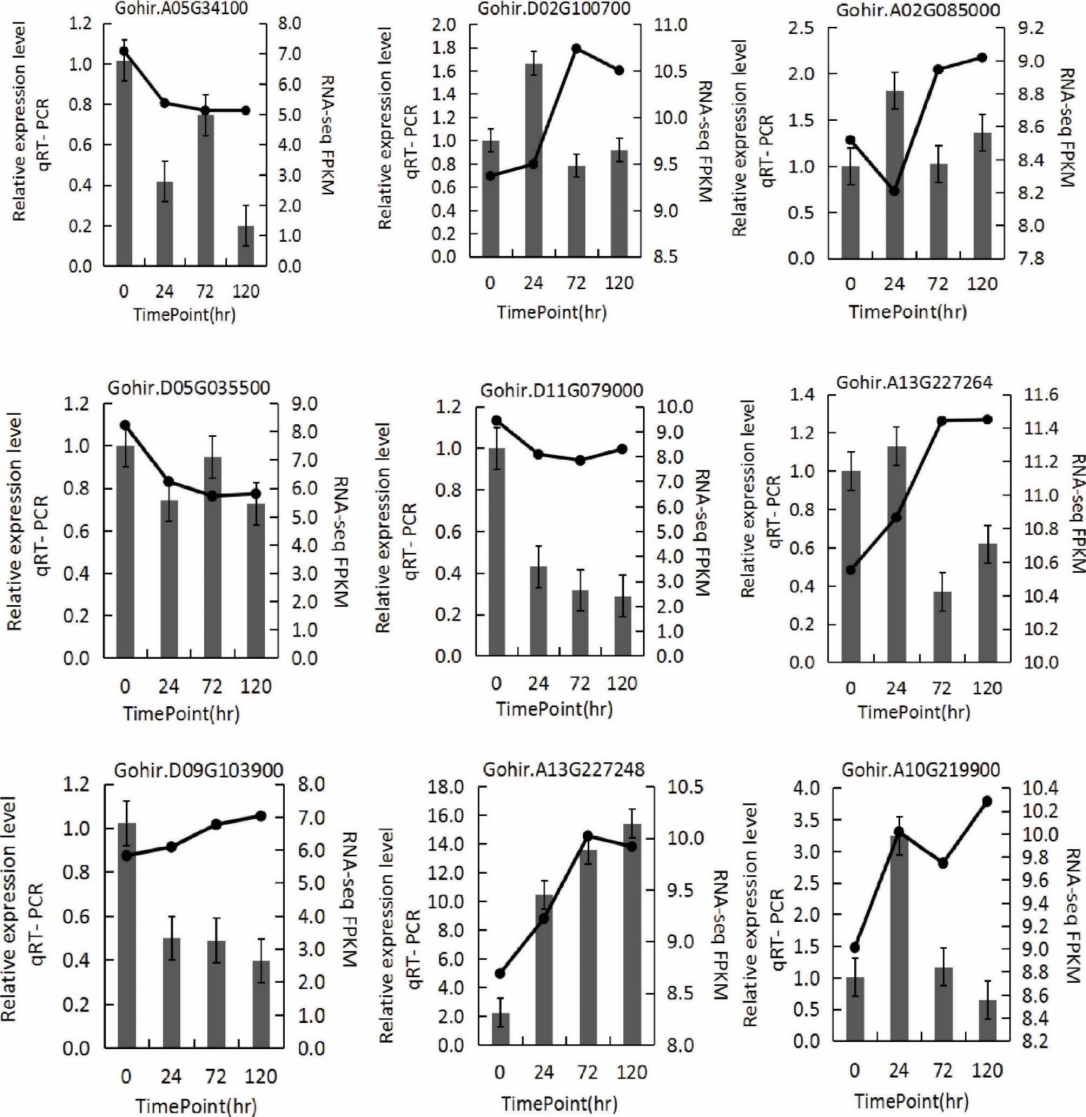
Expression pattern of RLCK VII genes in *G. hirsutum* “20B12” treated with *V*. *dahliae* “V991” measured using qRT-PCR. The line charts represent the gene expression trends in the RNA-Seq datasets, while the bar charts depict the gene expression level in qRT-PCR assays

To verify the involvement of RLCK-VII subfamily genes in response to *V. dahliae* V991, we analyzed 9 genes from *G. hirsutum* “20B12” using qRT-PCR (Fig. 7). Our findings showed differential regulation of RLCK-VII subfamily genes at different time-points of post *V. dahliae* inoculation, with some genes being up-regulated while others being down-regulated. For example, the expression levels of *Gohir.A05G034100*, *Gohir.D05G035500*, and *Gohir.D11G079000* were down-regulated after inoculation, and the expression levels of *Gohir.D02G100700*, *Gohir.A13G227264*, *Gohir.A13G227248*, *Gohir.A10G219900*, and *Gohir.A02G085000* genes were up-regulated after inoculation. These results were in agreement with RNA-Seq data, further backing the hypothesis that GhRLCK-VII subfamily genes are responsible for cotton resistance to *V. dahliae*.

### Function characterization of GhRLCK-VII genes in cotton respondence to *V. dahliae* through VIGS

**Fig. 8:**
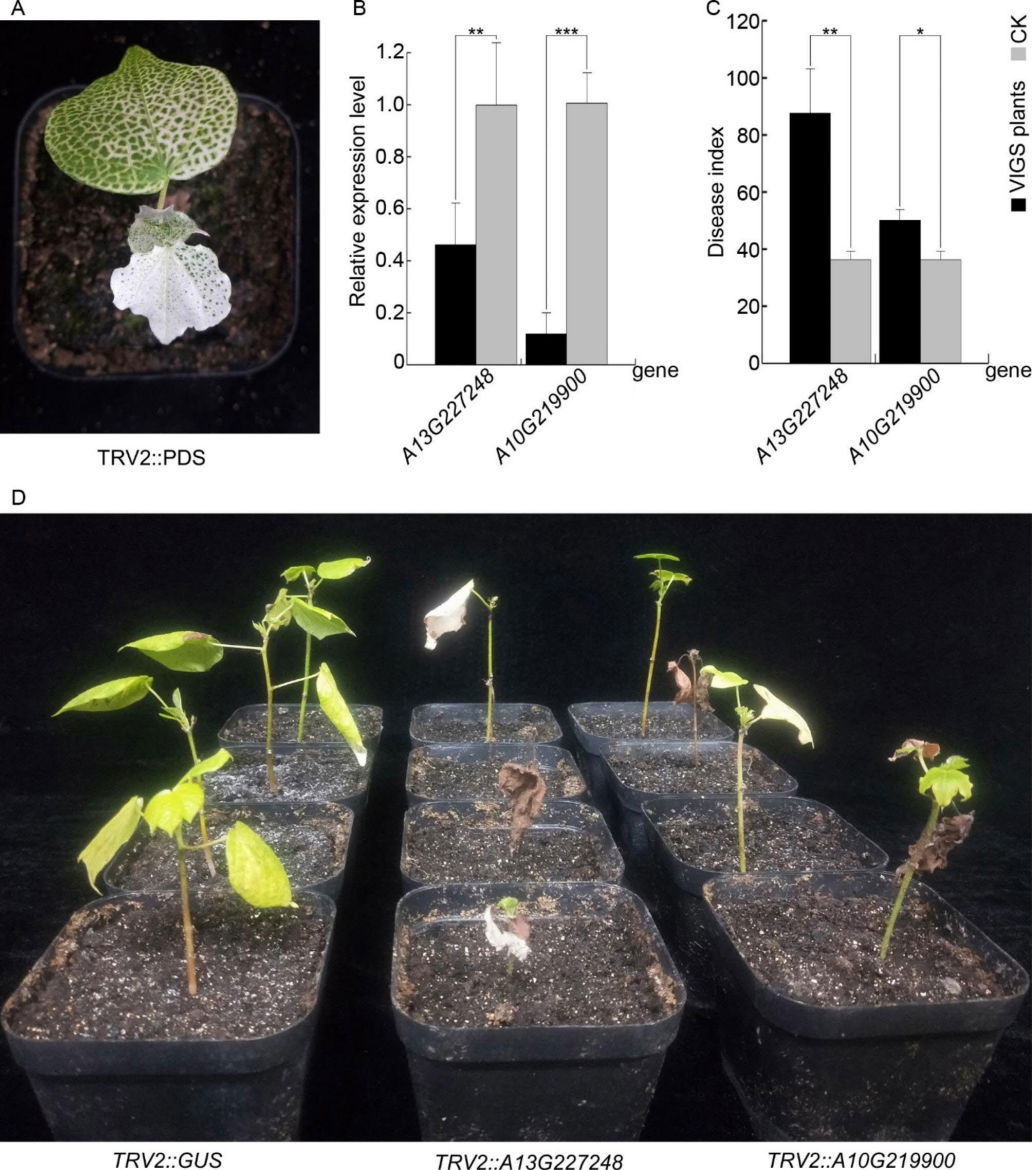
Reduced resistance of *G. hirsutum* “20B12” to *V*. *dahliae* “V991” after silencing *Gohir.A13G227248* and *Gohir.A10G219900*. **(A)** The bleached leaf phenotype of plants inoculated with *Agrobacterium tumefaciens* carrying TRV2::PDS. **(B)** qRT-PCR results after silencing *Gohr.A13G227248* and *Gohr.A10G219900*, respectively. **(C)** Disease index of *G. hirsutum* “20B12” at 60 days of *V. dahliae* “V991” infection. The Verticillium wilt disease index evaluation was conducted according to the Chinese Technical Specification for Evaluating Resistance to Verticillium wilt of Cotton (NY/T 2952–2016). **(D)** Phenotype of *G. hirsutum* “20B12” cultivar at 40 days of *V. dahliae* “V991” infection, showing severe necrosis and falling off of leaves compared to the control group

We utilized three different perspectives, including DNA (many *cis*-acting elements (respondence to stresses) in the 2000 bp sequences upstream), mRNA (significantly increased transcript abundance after inoculation with *V. dahliae*), and amino acid (phylogenetic classification and conserved domain), to infer that *Gohir.A13G227248* and *Gohir.A10G219900* may positively contribute to cotton resistance to *V. dahliae*. To further understand their function, we designed primers to amplify 342-bp and 345-bp fragments of these genes, respectively, and constructed TRV2::Gohir.A13G227248 and TRV2::Gohir.A10G219900 vectors by inserting them into the pTRV2 vector. After confirming their sequences, we transformed the constructs individually into *Agrobacterium*. After 14 days of agroinoculation, cotton seedlings infiltrated with TRV2::PDS exhibited consistently uniform bleaching (an albino phenotype) in newly sprouted leaves (Fig. 8a), while plants infiltrated with TRV2::Gohir.A13G227248 and TRV2::Gohir.A10G219900 exhibited reduced much lower expression of target genes compared with in the control plants infiltrated with TRV2::GUS (*P* < 0.01; Fig. 8b), indicating successful silencing of the target genes.

Subsequently, we inoculated cotton seedlings with *V. dahliae* and observed that the chlorosis spots arose in the leaf margins and veins after 7 days of inoculation. Compared with the control, the agroinoculated plants exhibited a higher number of chlorosis spots early. By about 40 days after inoculation, the plants agroinoculated with TRV2::Gohir.A13G227248 and TRV2::Gohir.A10G219900 showed more severe symptoms, including smaller but extensive chlorosis, gradual browning, wilting, and necrosis (Fig. 8c). By 60 days after inoculation, the leaves of agroinoculated plants developed more areas of dead brown tissues surrounded by larger areas of yellowing and showed a defoliating pathotype, and the stem’s vascular bundles were seriously browning. We calculated the disease index and observed that the cotton seedlings infiltrated with TRV2::Gohir.A13G227248 and TRV2::Gohir.A10G219900 were more susceptible to *V. dahliae*, as they showed a higher disease index than the control (*P* < 0.05, Fig. 8d). Additionally, the cotton seedling agroinoculated with TRV2::Gohir.A13G227248 exhibited more serious disease symptoms than the cotton seedling agroinoculated with TRV2::Gohir.A10G219900, with the disease index being 87.5% higher. Based on the combined results of qRT-PCR and the observed phenotype of VW, we inferred that *Gohir.A13G227248* and *Gohir.A10G219900* were essential for cotton resistance to *V. dahliae*.

## Discussion

Accumulating evidence has uncovered that RLCK-VII subfamily are actively involved in plant immune signaling pathways and essential in plant disease resistance, tolerance, growth, development, and other biological processes. In *Arabidopsis*, there are 46 RLCK-VII proteins that function in pattern-triggered immune signaling and confer plant resistance to pathogens [8]. However, a comprehensive research is still missing on RLCK-VII subfamily genes in *G. hirsutum*, which often encounters pathogen attacks.

In this study, we identified 72 GhRLCK-VII subfamily genes in *G. hirsutum* through bioinformatics analysis. As reported previously, most plant species have undergone large-scale whole-genome duplication events, including segmental duplication and tandem duplication, leading to a larger genome size. Allotetraploid *G. hirsutum* possesses much more and larger GhRLCK-VII genes than diploid *A. thaliana* (46 genes), indicating a positive correlation between genome and RLCK-VII subfamily size [3, 5].

Chromosomal segment duplication and tandem duplication provided raw genetic materials for new gene generation (gene gains) and contributed to phenotypic changes, facilitating species evolution [22]. Our collinearity analysis indicated that most duplication events resulted from chromosomal segmental duplication and all GhRLCK-VII genes were implicated in segment duplication. These results showed that following segmental duplication, the function of the newly paralogous genes remained relatively conserved during evolution, with preserved protein structures and functions. Additionally, our collinearity analysis found gene loss, pseudogene formation, or dissimilation. Dissimilation of duplicated genes may have facilitated the co-evolution of plants and pathogens, while gene loss or pseudogene formation slightly reduced gene redundancy. Overall, segmental duplication improved plants’ biological function and adaptability to diverse stresses.

*Ka*/*Ks* values were computed for pairwise collinear genes after MCScanX analysis to estimate selective pressure. The values indicated that the RLCK-VII genes in *G. hirsutum* underwent strict purification selection after expansion and retained primitive gene function. However, for a minority of GhRLCK-VII genes that lost their collinear counterpart due to gene loss, pseudogenization (defunction), or dissimilation, the related *Ka*/*Ks* values could not be computed.

To classify the GhRLCK-VII genes, we used *Arabidopsis* RLCK-VII subfamily, which has been systematically investigated [8], as the reference and grouped them into nine groups according to the phylogenetic tree (Fig. 1). Our classification revealed that 5 genes did not fall into any group. Compared to *Arabidopsis*, the sizes of groups VII-1, VII-4, VII-5, and VII-6 were expanded 2−3 times in *G. hirsutum*. For example, *AtPBS1* in the group VII-1 was highly homologous to *Gohir.A01G021700*, *Gohir.A01G021800*, and *Gohir.D01G020200* in terms of phylogenetic relationship. In terms of amino acid sequences, motif arrangements, and conserved subdomains (Table S9), RLCK-VII members within a group (intragroup) were similar to each other but different (intergroup) from those in other groups. Moreover, all genes within a phylogenetic group possess a similar exon-intron structure, which may be evidence of their derivation from a common ancestor [23].

Our identification using local HMMER search and online SMART analyses (http://smart.embl.de) indicated that 72 members of the RLCK-VII subfamily had two conserved domains, PKinase-Tyr (pfam07714) and PKinase (pfam00069), which overlapped each other. However, local InterProScan analyses showed that every member contained only one domain, with 44 and 28 members containing pfam07714 and pfam00069, respectively. This discrepancy may be due to differences in the algorithms implemented by each program, highlighting the need to integrate results from different programs. Additionally, local InterProScan analyses showed that prosite PS50011 (protein kinase domain) was present in 72 members, PS00107 (Protein kinases ATP-binding region signature) and PS00108 (Serine/Threonine protein kinases active-site signature) were present in 63 and 62 members, respectively, and 53 members contained three prosites. Furthermore, local InterProScan analyses also showed 19 members contained a SMART00220 (catalytic domain). These abundant sites implied the diverse roles of members of the RLCK-VII subfamily.

The conserved kinase active sites create a stable milieu for RLCKs to execute their function in the cytoplasm. Previous studies indicated that mojority RLCK kinase active sites are aspartic acid (D). Fan et al. [6] showed that the kinase activity sites for RLCK-VII subfamily are ‘IYR**D**IKASNIL’, and every subfamily in RLCK harbors specific kinase activity sites. However, we found 17 kinds of activity sites (excluding ‘IYR**D**IKASNIL’) in the GhRLCK-VII subfamily, among which the predominant sites are ‘IYR**D**FKTSNILL’ and ‘IYR**D**FKASNILL’, which are not present in other subfamilies in the RLCK family [6]. The sequence pattern of activity sites in GhRLCK-VII is ‘IYR**D**[FL]K[TSA]SNIL’ present in the aforementioned motif-3.

The GDK motif is a cleavage motif, where cleavage occurs during plant immune response, i.e., AthPBS1 is cleaved by the effector AvrPphB. We found that 36 (50%) GhRLCK-VII members harbor the ‘GDK’ motif. This cleavage exposes the SEMPH motif in the C-terminal loop, that is recognized and bound by the NLR protein family member RPS5, activating RPS5 and subsequently inducing a hypersensitive response (HR) [24]. We found that Gohir.A01G021700 and Gohir.D01G020200 harbor an STRPH motif rather than the SEMPH recognition motif, consistent with TaPBS1 in wheat [24]. Sun et al. [24] found that recognition of PBS1 by RPS5 requires a negatively charged amino acid residue at the “E” position of the SEMPH motif. Therefore, our results support the highly diverse resistance recognition motifs in PBS1 orthologs.

The examination of PBS1 orthologs in various plant species revealed that all the orthologs contained several preserved subdomains of PBS1 kinase [25]. Analysis using the program PS_scan revealed the presence of several subdomains in GhRLCK-VII members, such as GxGxxG, K, E, DxxxxN, DFG, APE, DxxxxG and R, showing similar sequence patterns to ‘**G**E**G**GF**G’**, ‘[VI]A[VI]**K**’, ‘[EQ]xxx**E**xxxL’, ‘**D**xKxx**N**’ (which overlapped with ‘IYR**D**[FL]K[TSA]S**N**IL’), ‘DFG’, ‘[**A**D]**P**[**E**DG]Y’, ‘**D**[VI][YWF]xx**G**[VI]’, and ‘**R**Px[MI]’, respectively (Table S9). This indicates the conservation and/or diversity between GhRLCK-VII members and PBS1 orthologs. Moreover, MEME analyses indicated that motif-1 contained subdomains DFG, GDK, APE, and DxxxxG; motif-2 contained subdomains K and E; motif-4 contained subdomain GxGxxG [25]. These conserved kinase subdomains and motifs may be critical to directing immune response of GhRLCK-VII members, allowing them to interact with a various downstream proteins and giving rise to diverse responses depending on the effector’s nature [25].

The RNA-Seq and qRT-PCR assays demonstrated that GhRLCK-VII genes exhibited diverse tissue-specific expression patterns. For instance, *Gohir.A12G166000* was overexpressed in anther, while *Gohir.A06G147500* was highly expressed in 25 DPA fiber. The expression of *Gohir.A11G062700* was high in most tissues but decreased after inoculation with *V. dahliae*, implying that RLCK-VII genes may contribute to specific growth and development stages, in line with several previous investigations [2, 8].

Genes in the same phylogenetic group may contribute redundancies that originated from whole genome duplication events. However, whether they differ in spatiotemporal expression patterns has not been completely investigated. We found that *Gohir.A10G204300*, *Gohir.D10G212300*, and *Gohir.D13G232700* of the RLCK-VII-4 group were highly expressed in some tissues (torus, bracts, sepals, petals, filaments, anthers, ovules, and fibers), but lowly expressed in others (roots, stems, and leaves) and leaves after *V. dahliae* infection. Reversely, both *Gohir.A05G095400* and *Gohir.A13G227264* in the RLCK-VII-8 group were highly expressed in anther and 10 DPA fiber, respectively. Therefore, redundant and phylogenetically related members of the RLCK-VII subfamily may have either similar expression patterns, resulting in biological promotion, or different and specific expression patterns, reducing redundancy.

Investigating tissue-specific expression profiles offers valuable information regarding the contribution of GhRLCK-VII genes in plant growth and development. Furthermore, our understanding of the potential involvement of GhRLCK-VII genes in plant responses to biotic and abiotic stresses remains limited. Yang et al. [26] showed that *Gbvdr6*, an RLK gene of *G. barbadense*, enhanced the resistance to *V. dahliae* in transgenic *Arabidopsis* and cotton plants. *GhWAK7A*, a wall-associated kinases (WAKs) gene belonging to RLKs, also enforced cotton defense against *V. dahliae* infections [27]. RLCK-VII members are generally regulated by various pathogen effectors [8]. In this study, analyses of the RNA-Seq datasets (SRP192537 and SRP328396) from cotton inoculated by *V. dahliae* revealed that some genes were up-regulated while others were down-regulated. Our qRT-PCR assays provided convincing supplements and validation, followed by gene knockdown through VIGS and symptoms phenotyping. The expression profile after *V. dahliae* inoculation and VIGS assays of *Gohir.A13G227248* (ortholog of *AtPBL37*) and *Gohir.A10G219900* (ortholog of *AtBIK1*) indicated that GhRLCK-VII genes were involved and might play certain regulatory roles in responding to *V. dahliae*. Veronese et al. [9] reported that BIK1 modulated various cellular factors necessary to defense responses against pathogen infection and normal root hair growth, connecting defense response regulation with growth and development. Our results are in line with the reported role of BIK1 in *Arabidopsis*.

## Conclusion

Our comprehensive identification revealed 72 GhRLCK-VII genes in *G. hirsutum*. Characterization of their DNA and amino acid sequences provides insights into their location, composition, evolution, conservation, and diversity. RNA-Seq datasets and qRT-PCR unveiled diverse spatiotemporal expression patterns, and VIGS assays further indicated the potential impacts of GhRLCK-VII genes on cotton defense responses against pathogens and on plant growth and development. Our findings open novel avenues for further studying disease response pathways and enhance our comprehension of GhRLCK-VII functions.

### Methods

#### Identification of RLCK-VII gene subfamily and phylogenetic tree construction

The genome sequences, coding sequences (CDS), general feature format version 3 (GFF3) annotation information, and protein sequences of *G. hirsutum* were retrieved from the CottonGen database (https://www.cottongen.org/cottongen_downloads/Gossypium_hirsutum/ UTX-TM1_v2.1/) [28]. Additionally, 46 protein sequences of *A. thaliana* RLCK-VII subfamily members were retrieved from the *A. thaliana* database (https://www.arabidopsis.org) [8]. The amino acid sequences of the two conserved Pkinase (pfam00069) and Pkinase-Tyr (pfam07714) domains were acquired from the Pfam v35.0 database (https://pfam.xfam.org).

To uncover members of the RLCK-VII subfamily in *G. hirsutum*, the protein sequences of AtRLCK-VII members were utilized as queries and aligned with all protein sequences of *G. hirsutum* by running BLASTP locally. The HMMER v3.3.1 software was used to build the HMM profile based on the two conserved domains, and the proteins containing both conserved domains were identified using hmmsearch. The candidate protein sequences of RLCK-VII subfamily were further validated by screening the intersection of BLASTP subjects and HMMsearch results and submitted to the SMART database (http://smart.embl.de/), local InterProScan, Phobius (https://phobius.sbc.su.se/), and DeepTMHMM (https://dtu.biolib.com/DeepTMHMM) to pinpoint proteins lacking transmembrane domains and ectodomains. Subsequently, subcellular localization was projected using Plant-mPLoc (http://www.csbio.sjtu.edu.cn/bioinf/plant-multi/) [29]. Proteins containing kinase domains lacking transmembrane domains and ectodomains were ultimately identified as members of the *G. hirsutum* RLCK-VII subfamily.

To analyze the relationship between *G. hirsutum* RLCK-VII members and 46 members of *A. thaliana*, their amino acid sequences were analyzed using the ClustalX program for multiple sequence alignment. Subsequently, a phylogenetic tree was generated using the neighbor-joining (NJ) method with a bootstrap value of 1000 via MEGA software [30]. Finally, based on the phylogenetic tree, *G. hirsutum* RLCK-VII members were categorized into several groups.

### Chromosome localization, physicochemical properties, and signal peptide of GhRLCK-VII members

The chromosome length and physical location information of genes on *G. hirsutum* chromosomes was extracted from the GFF3 annotation file. The positions of GhRLCK-VII genes on chromosomes were graphically displayed using MapChart. The amino acids count, theoretical isoelectric points (pI), molecular weight, protein instability index, aliphatic index, and grand average of hydrophobicity (GRAVY) of GhRLCK-VII proteins were calculated using the ProtParam (http://web.expasy.org/ProtParam/) [31]. Signal peptides were projected using SignalP-6.0 (http://www.cbs.dtu.dk/services/SignalP) [32].

### Gene structures, domains, and motifs of GhRLCK-VII subfamily

The conserved motifs of GhRLCK-VII members were determined employing the online software MEME 5.4.1 (https://meme-suite.org/meme/tools/meme) with a maximum search for ten motifs [33]. The NCBI Batch CD-Search (https://www.ncbi.nlm.nih.gov/cdd/) was used to project protein structure domains of the GhRLCK-VII subfamily [34]. The exon-intron information was garnered from the genome annotation file (Ghirsutum_527_v2.1.gene.gff3). The phylogenetic tree, motif arrangement, gene structure, and component domains of GhRLCK-VII subfamily members were visualized using TBtools software [35].

### Collinearity and evolutionary relationship among GhRLCK-VII genes

To investigate gene duplication during evolution within species, the collinearity between genes and genes’ physical location on chromosomes of GhRLCK-VII subfamily members was analyzed using MCScanX and visualized using Circos [35, 36]. Non-synonymous mutation rate (*Ka*), synonymous mutation rate (*Ks*), and *Ka*/*Ks* value of selection pressure were obtained using the KaKs_Calculator [37].

### Exploration of *cis*-acting elements

The upstream 2000 bp DNA sequences of GhRLCK-VII genes were searched for *cis*-acting elements using PlantCARE (http://bioinformatics.psb.ugent.be/webtools/plantcare/html/) [38].

### Expression profiles of GhRLCK-VII genes

#### Transcriptome data (RNA-Seq) analysis

To study GhRLCK-VII expression profiles in various tissues and at distinct time-points following *V. dahliae* inoculation, three sets of RNA-Seq data (SRP166405, SRP192537, and SRP328396) were garnered from NCBI SRA database. The short read sequences were mapped to *G. hirsutum* genome using HISAT2, and GhRLCK-VII expression levels under different temporal and spatial conditions were calculated using DESeq2. The RNA-Seq project SRP166405 included 11 tissues: root, stem, leaf, torus, bract, sepal, petal, filament, anther, ovule at 0-, 1-, 3-, 5-, 10-, 15-, 20-, and 25-days post anthesis (DPA), and fiber at 10, 15, 20, and 25 DPA. The RNA-Seq project SRP192537 included several samples collected at 6 h, 12 h, 24 h, 48 h, and 72 h after *V. dahliae* inoculation. The RNA-Seq project SRP328396 included samples harvested at 0 h, 24 h, 72 h, and 120 h after inoculating Xinluzao-36 (susceptible) and Zhongzhimian-2 (tolerant) cultivars with two *V. dahliae* strains [39].

#### Inoculation with *V. dahliae* and qRT-PCR validation

Nine genes were selected to verify the expression profiles of GhRLCK-VII subfamily genes using qRT-PCR assays with specific primers (Table S1) designed using Oligo 7.60 and synthesized by the Xian Qingke Biotechnology Company. In detail, highly virulent *V. dahliae* strain “V991” from our laboratory was grown on potato dextrose agar (PDA) medium in the dark at 25 °C for about 10 days. After rinsed with sterile distilled water, *V. dahliae* was adjusted 1 × 10^6^ conidia/mL. Meanwhile, *V. dahliae* tolerant *G. hirsutum* cv. “20B12” was grown in an incubator. At the 3-leaf stage, the cotton seedlings were infected with *V. dahliae* using the dipping root method, transplanted to an incubator with new soil, and cultured for different durations [40].

The seedling roots were gathered at 0 h (CK), 24 h, 72 h, and 120 h post-inoculation with 3 or more independent plants. Total RNAs were extracted using the RNA Prep Pure Plant Kit (Tiangen Biochemical Technology (Beijing) Co., Ltd.) as per the manufacturer’s instructions. RNA concentration and integrity were measured using NanoDrop and 1.2% agarose gel electrophoresis, respectively. cDNAs were generated using the HiFi Script gDNA Removal cDNA Synthesis Kit (Kang Wei Century Company) and subjected to qRT-PCR assay in a 20-µL reaction system on a QuantStudio 7 Flex Real-time Fluorescence Quantitative System with *UBQ7* as the internal control. The reaction was proceeded at pre-denaturation at 95 °C for 10 min prior to 40 cycles of 95 °C for 15 s, 60 °C for 1 min, and 72 °C for 15 s. The relative gene expression was calculated using the 2^−ΔΔCt^ method.

### Virus-induced gene silencing (VIGS) assay and symptom phenotyping

RNAs were isolated from “20B12” leaves, converted to single-strand cDNA, and used to amplify *Gohir.A13G227248* and *Gohir.A10G219900* using primers with *Kpn*I and *Xba*I restriction sites and overhangs designed using Oligo 7.60 software (Table S1). After treated with with *Kpn*I and *Xba*I, the generated fragments were cloned into TRV2 vector. After sequencing verification, the favorable plasmid was prepared and transformed into Agrobacterium GV3101. The agrobacterium strains carrying TRV2::Gohir.A13G227248, TRV2::Gohir.A10G219900, TRV2::PDS, TRV2::GUS, and TRV1 were grown in YEB liquid medium supplemented with kanamycin, rifampicin and gentamicin at 28 °C at 200 rpm. After culturing for 10–12 h when OD_600_ value reached 1.0, bacteria were precipitated, resuspended in an Agrobacterium induction buffer with 20 g/L sucrose, 5 g/L MS salts, 1.95 g/L MES, pH 5.6, and placed at 22 °C for 2 h. The TRV1 and TRV2::gene cultures were combined at a 1:1 (vol/vol) ratio and inoculated into the abaxial side of the cotyledons of 14-day-old leaf-free *G. hirsutum* cv. “20B12” seedlings. The inoculated plants were incubated for 24 h in the dark, followed by a ‘16 h light/8 h dark’ photoperiod. When the cotton seedlings infiltrated with TRV2::PDS showed a bleached leaf phenotype, leaf RNAs were extracted and used for qRT-PCR assay to evaluate the silencing efficiency. Meanwhile, cotton seedlings’ roots were dip-inoculated with *V. dahliae* “V991” conidia suspension. After dip-inoculation, the cotton seedlings were transplanted and grown in an incubator with new soil to observe symptoms. At 35 days of post-dip-inoculation, the number of diseased plants and the degree of disease were recorded to calculate the disease index.

### Electronic supplementary material

Below is the link to the electronic supplementary material.

Supplementary Material 1

Supplementary Material 2

## Data Availability

All data generated or analyzed during this study are included in this published article and its supplementary information files. The datasets analysed during the current study are available in the CottonGen database (https://www.cottongen.org/cottongen_downloads/Gossypium_hirsutum/UTX-TM1_v2.1/).
