# Supplementary material for: Genome-wide identification of GhRLCK-VII subfamily genes in Gossypium hirsutum and investigation of their functions in resistance to Verticillium wilt
Source: BMC Plant Biol. 2023 Sep 11;23:421. doi: 10.1186/s12870-023-04435-0 (PMC10494381; doi:10.1186/s12870-023-04435-0)
Supplement: Supplementary file 2 — Supplementary Material 2 [file 12870_2023_4435_MOESM2_ESM.docx]

**Supplementary Materials**

**Table S1**. Primers for gene cloning (VIGS) and qRT-PCR assays. The underscored nucleotide sequences indicate the recognition sites for restriction enzymes.

**Table S2**. Identification of GhRLCK-VII members using various software, including local HMMER, local BLASTP, online SMART database (http://smart.embl.de/), local InterProScan, online Phobius (https://phobius.sbc.su.se/), online DeepTMHMM (https://dtu.biolib.com/DeepTMHMM), and online Plant-mPLoc (http://www.csbio.sjtu.edu.cn/bioinf/plant-multi/).

**Table S3**. Classification of GhRLCK-VII according to the phylogenetic tree containing AtRLCK-VII and GhRLCK-VII members reported by Rao Shaofei, et al. Plant Physiology, 2018, 177(4): 1679–1690. [https://doi.org/10.1104/pp.18.00486](https://doi.org/10.1104/pp.18.00486.).

**Table S4**. Information on the 10 motifs present in GhRLCK-VII proteins as predicted using software MEME.

**Table S5**. Motif arrangement in GhRLCK-VII proteins, including order and position.

**Table S6**. Collinearity and chromosomal segmental duplication of GhRLCK-VII genes, along with their Ka/Ks ratios.

**Table S7**. Number of cis-acting regulatory elements in the 2000 bp sequences upstream of GhRLCK-VII genes, as searched using online PlantCARE (http://bioinformatics.psb.ugent.be/webtools/plantcare/html/)).

**Table S8**. GhRLCK-VII expression levels based on RNA-Seq datasets SRP166405, SRP192537, and SRP328396.

**Table S9**. Arrangement of conserved kinase subdomains in GhRLCK-VII proteins.
